# The pandemic agreement: Achieving an African win for health security inequity

**DOI:** 10.4102/jphia.v15i1.618

**Published:** 2024-05-21

**Authors:** Nicaise Ndembi, Nebiyu Dereje, Fifa A. Rahman, Benjamin Djoudalbaye, Aggrey Aluso, Nina Schwalbe, Tajudeen Raji, Mosoka P. Fallah, Sofonias K. Tessema, Mohamed Moussif, Sultani Matendechero, Olive Shisana, Alain N. Ngongo, Jean Kaseya

**Affiliations:** 1 Africa Centres for Disease Control and Prevention (Africa CDC), Addis Ababa, Ethiopia; 2 Matahari Global Solutions, Kuching, Sarawak, Malaysia; 3 Pandemic Action Network, Resilience Action Network Africa, Nairobi, Kenya; 4 Spark Street Advisors, New York, United States of America; 5 Casablanca International Airport, Ministry of Health, Casablanca, Morocco; 6 State Department for Public Health and Professional Standards, Ministry of Health, Nairobi, Kenya; 7 Department of Psychiatry and Mental Health, University of Cape Town, Cape Town, South Africa

The coronavirus disease 2019 (COVID-19) pandemic has tested the resilience of global health systems and exposed their frailty and disparities across health systems worldwide, particularly in Africa.^1^ As pandemic responses kicked in, the introduction and deployment of COVID-19 vaccines, therapeutics, and diagnostics were woefully inadequate, and in the aftermath demonstrated a clear need for reform. Particularly in the earlier days of the disease and vaccine introduction, the response was characterised by vaccine nationalism, a lack of transparency in data sharing, and an overall lack of inclusivity and Global South integration in global response mechanisms. As a result of these and other inequities, the global health community raised concerns about global health security frameworks, such as the inability of the International Health Regulation (IHR) to prepare countries for a global health emergency. Criticisms centred, inter alia, around the IHR’s limitations on timely investigation of public health emergencies, an insufficiently robust compliance system, scarcity of resources, and conflicting advice.^2,3^

Cognisant of these gaps in the IHR, the World Health Organization (WHO) formed an Intergovernmental Negotiating Body (INB) to draft a new legally binding Pandemic Agreement and to facilitate the conclusion of member state negotiations by the upcoming 77th World Health Assembly (WHA) in May 2024.^4^ Underlining the Agreement are notions that this new agreement should fill policy gaps in the IHR and ensure equity worldwide. At the time of writing, WHO member states have been engaging in textual negotiations over crucial issues of the Pandemic Agreement, including what has been referred to as the ‘heart of the political bargain’ of the Agreement, that is, pathogen access and benefit sharing (PABS), as well as Research and Development (R&D) conditionalities, financing and technology transfer. During the two rounds of negotiations, the member states agreed on various issues such as governance mechanisms, compliance and implementation, workforce, and R&D, among others. However, they failed to reach consensus on critical issues such as PABS, pandemic prevention and one health, and pandemic financing. The WHO announced that the member states will continue the negotiation on the articles to reach into agreement before the 77th WHA.^5^

Access to PABS – the rapid sharing of pathogens and their sequence data and equitable and timely access to countermeasures for pandemic preparedness and response – is particularly critical. Rapid sharing of pathogen data must be reciprocated with multilateral benefits. This could be done, for example, by allocating a percentage of production to developing countries or financial contributions towards capacity-building and complementing it by other types of in-kind and voluntary contributions such as technology transfer. However, there is opposition, primarily from high-income countries towards many different elements of a PABS system ranging from claims that electronic tagging would not be technically feasible, to assertions that current data-sharing platforms, like the Nagoya Protocol, are already working well enough for Pandemic Prevention Preparedness and Response (PPPR).^6^ However, the COVID-19 pandemic highlighted the imbalances in equity between countries during global public health emergencies. As an example, South African researchers shared genomic sequence data for the Omicron variant, but the vaccines developed by pharmaceutical companies in high-income countries using these sequences were largely inaccessible to the majority of Africans. The country was also penalised for sharing (e.g., travel bans).^7^ The rapid sharing of pathogens and their sequence data are also critical for pandemic preparedness and response but because of developments in international law in recent years, pathogen sharing has been impeded. We strongly believe that the PABS system is the most critical element of the agreement that can provide a guarantee to ensure safety and equity for all, whether high-income or low-income countries. After taking cognisance of this fact, the Ministers of Health of African Union Member States held a meeting on 27 April 2024, recalling the negotiators of the Pandemic Agreement to ensure equity through the multilateral PABS system, and financial investments that support sustainable and geographically diversified production of countermeasures.^8^

The essentiality of having robust governance, compliance management, and implementation systems in the Pandemic Agreement is unarguable. However, we believe that these should be constructed in a manner to balance both the need to inspire confidence in the governance and compliance systems and the complementarities between the Agreement and the IHR while ensuring that African nations are not overburdened by reporting bureaucracy.^9,10^ We remain open to debates around whether the compliance and implementation committees should be conjoined or exist as two separate entities, although, arguably, the functions of the two are different: compliance to ensure whether specific obligations are adhered to and whether country reports are accurate, timely and complete and implementation to monitor and support operationalisation in countries. Good governance is key to the treaty overall and its individual components, such as any mechanisms set up to facilitate financing, PABS, and strengthening the supply chain. Donors cannot have an oversized representation in decision-making.

To negotiate and adopt the agreement, African countries must translate the lessons learned from the COVID-19 pandemic. When COVID-19 vaccines became available, they quickly became a game changer, reducing transmission, disease severity, and mortality. However, Africa was largely left behind, with few vaccines available and much lower vaccination coverage. The African Union Commission and Africa Centres for Disease Control and Prevention (Africa CDC) initially dealt with this challenge through a conventional approach, in which vaccines would be purchased through the COVID-19 Vaccine Global Access (COVAX) facility (part of the Access to COVID-19 Accelerator or the ACT-A).^11^ Access to COVID-19 Accelerator had serious and life-costing pitfalls: it took too long to raise the financing, vaccine deployment was delayed by issues of export bans and other geopolitical tensions, the diagnostics and therapeutics pillars did not meet their targets, and the health systems connector pillar did not operationalise adequately and failed to meet its mandate, compromising critical last mile capabilities. According to the ACT-A external evaluation, a similar platform for future pandemics should have better coordination on R&D so that there should be available contingent funding on Day Zero of the next pandemic, and there should be a ‘strong representation of regional actors’ in the governance structure and a stronger emphasis on technology transfer.^2^ The Pandemic Agreement must address some of these barriers.

On the margins of the 2023 Brazil, Russia, India, China and South Africa (BRICS) summit, a meeting co-hosted by the Africa CDC, the Ministry of Health in South Africa, Africa Health Business and the South African Chapter of the BRICS Business Council highlighted the AU and BRICS framework of cooperation for the pandemic prevention, preparedness, and response (PPPR). This summit recognised the relevance of the ‘Johannesburg Process’, which aims to ensure equitable and timely access to medical countermeasures. However, we believe that the governance of such a mechanism must be more inclusive to capitalise on indigenous expertise in delivering these tools. The summit further emphasised the merit of opening up the market to all pharmaceutical manufacturers in the African Union (AU) and BRICS regions, and the heads of state endorsed this during the AU summit, which created a $50 billion market for manufacturers. The bold progress made by the AU and Africa CDC to ensure people’s health security must be supported by similar bold commitments on regional manufacturing, technology transfer, and R&D conditionalities in the Pandemic Agreement. The Pandemic Agreement must ensure the sustainable and geographically diversified production of countermeasures – Africa must be engaged in production rather than being a mere consumer of the products.

The relevance of the Pandemic Agreement for African countries is indisputable. With more than 160 emerging and re-emerging public health emergency events in a year and with climate change further exacerbating these, Africa critically demands global collaboration to ensure future PPPR, and a robust agreement that serves Africa’s interests is essential to achieve this purpose. The role of African leadership in this endeavour is indispensable.^12^ Our support for a legally binding Agreement does not negate our belief that pitfalls in the draft need to be addressed in a transparent and win-win approach to reach a sustainable and enforceable agreement. The agreement must ensure equity in possible strong terms, and in terms that do not allow for derogation based on conditional language. Equity, integrated in all parts of the text, is the only way to prevent the wide health and economic implications of a global pandemic on our African countries, the rest of the Global South, and high-income nations alike.
